# NADES-Mediated Deposition of Potential Biomimetic Drug-Loaded Polypyrrole on Biomedical Ti20Zr5Ta2Ag

**DOI:** 10.3390/biomimetics10090568

**Published:** 2025-08-25

**Authors:** Radu Nartita, Florentina Golgovici, Ioana Demetrescu

**Affiliations:** 1 Department of General Chemistry, Faculty of Chemical Engineering and Biotechnologies, National University of Science and Technology Politehnica Bucharest, Splaiul Independentei Street, No. 313, 060042 Bucharest, Romania; radu_sorin.nartita@upb.ro (R.N.); ioana.demetrescu@upb.ro (I.D.); 2 Academy of Romanian Scientists, 3 Ilfov, 050094 Bucharest, Romania

**Keywords:** natural deep eutectic solvent, polypyrrole, Naproxen, high-entropy alloy, corrosion protection, drug release

## Abstract

A natural deep eutectic solvent (NADES)-based electropolymerization strategy was developed to deposit polypyrrole (PPy) and Naproxen-doped PPy films onto a biomedical Ti–20Zr–5Ta–2Ag high-entropy alloy. Using cyclic voltammetry, chronoamperometry, and chronopotentiometry, coatings were grown potentiostatically (1.2–1.6 V) or galvanostatically (0.5–1 mA) to fixed charge values (1.6–2.2 C). Surface morphology and composition were assessed by optical microscopy, SEM and FTIR, while wettability was quantified via static contact-angle measurements in simulated body fluid (SBF). Electrochemical performance in SBF was evaluated through open-circuit potential monitoring, potentiodynamic polarization, and electrochemical impedance spectroscopy. Drug-release kinetics were determined by UV–Vis spectrophotometry and analyzed using mathematical modelling. Compared to uncoated alloy, PPy and PPy–Naproxen coatings increased hydrophilicity (contact angles reduced from ~31° to <10°), and reduced corrosion current densities from 754 µA/cm^2^ to below 5.5 µA/cm^2^, with polarization resistances rising from 0.06 to up to 37.8 kΩ·cm^2^. Naproxen incorporation further enhanced barrier integrity (R_coat_ up to 1.4 × 10^11^ Ω·cm^2^) and enabled sustained drug release (>90% over 8 days), with diffusion exponents indicating Fickian (n ≈ 0.51) and anomalous (n ≈ 0.67) transport for potentiostatic and galvanostatic coatings, respectively. These multifunctional PPy–Naproxen films combine robust corrosion protection with controlled therapeutic delivery, supporting their potential biomimetic role as smart coatings for next-generation implantable devices.

## 1. Introduction

At the beginning of the century, in 2004 [1], when the first synthesis of a HEA alloy (high-entropy alloy) took place, this new advanced material offered the hope of obtaining remarkable mechanical properties in extreme conditions [2], breaking the property limits of traditional alloys to enable their use in top future industrial applications [3]. Their initial formulation, the so-called Cantor alloy CrMnFeCoNi, was a mixture of five elements in equal atomic ratios, unlike traditional alloys that typically focus on one or two main elements. The composition of the Cantor alloy exhibits very good mechanical and anticorrosion behavior even at elevated or cryogenic temperatures [4,5]. New strategies have been explored to achieve the desired properties based on the HEA design concept, which permits extensive micro- and nanostructural manipulation and optimization procedures [6,7,8,9].

These strategies can be divided into component research design, structural design, and post-processing procedures [10]. By including new elements, the first category introduced Ti-based HEAs such as TiZrHfNbTa and its subvariants TiZrNbTa, TiZrHfTa, and TiZrHfNb [11,12]. Combining impressive corrosion resistance and mechanical properties with a better cell response compared to pure Ti, Ti-based HEAs are now recognized as versatile biomaterials in various bioapplications [13,14]. It should be mentioned that different compositions with improved performance have been introduced and investigated in the last decade [15,16]. One of them, 73Ti-20Zr-5Ta-2Ag, was obtained and characterized [17,18] in a large project for developing more implant alloys and was more recently coated [19,20] to extend its capability under simulated service-life conditions. Members of our research group participated in all steps of this challenge, yielding interesting results in integrative physical–chemistry research of materials and biology.

Deep eutectic solvents (DES) and natural deep eutectic solvents (NADES) have also emerged as versatile media for the electrodeposition of polymers, metals and hybrid structures, since they dissolve both inorganic precursors and bioactive molecules while offering a wider electrochemical window than water and suppressing unwanted side-reactions [21,22]. Films formed in DES/NADES stand out for their high adhesion, compact microstructure and superior electrochemical stability compared to aqueous deposits, a result of the dense hydrogen-bond network that blocks aggressive species and shifts corrosion potentials to more noble values by suppressing both anodic and cathodic processes [23].

By tuning the hydrogen-bond donor (HBD) to acceptor (HBA) ratio and introducing bio-derived components, one can readily adjust viscosity and conductivity, facilitate uniform film growth, and embed therapeutic ions in situ to prime the coating for controlled release or antibacterial action [24,25].

The synergy between DES/NADES chemistry and film architecture reduces porosity and enhances adhesion, while in-process incorporation of heteroatoms or functional ions produces coatings that combine anticorrosive barriers with antibacterial or osteoinductive activity. Controlled nanostructure further enables sustained therapeutic release or antimicrobial-peptide anchoring, making them multifunctional platforms for next-generation implants [26]. Similar surface decoration of Ti–6Al–4V with oligopeptides has been shown to enhance osteoblastic differentiation and mitigate inflammatory responses [27].

Moreover, bioactive coatings on multi-principal-element alloys thus combine robust corrosion barriers with favorable biological responses. Cu or Ag enriched films exhibit pronounced antibacterial activity, while refractory elements promote spontaneous passivation and stable oxide formation, limiting release of potentially toxic ions [28,29,30].

In particular, NADES have been proposed as “green” electropolishing electrolytes, offering high ionic conductivity and thermal stability that favor uniform polymer growth [31], and have enabled polypyrrole (PPy) films with controlled morphology and in-process pharmaceutical incorporation [32].

Inspired by the research on polymeric films successfully tested and promoted in biomedical engineering for various applications [33,34,35], the present paper is an original study of a new potential biomimetic polymeric coating on 73Ti-20Zr-5Ta-2Ag alloy with Naproxen incorporated.

Biomimetics in implant research refers to strategies inspired by natural biological systems, encompassing both structural design (e.g., surface topographies that promote cell adhesion) and functionalization (e.g., controlled release of bioactive molecules) [36]. Such strategies aim to enhance implant integration by uniting protective and therapeutic functions within a single system. Within this framework, conductive polymers like polypyrrole are notable for offering stability and controlled drug delivery.

Naproxen was chosen due to its anti-inflammatory and analgesic properties, which can reduce the risk of post-implantation inflammation and pain management [37]. Its aromatic and carboxylate groups favor stable incorporation in polypyrrole, enabling controlled release at the implant site. Our approach uniquely combines a green NADES electrolyte, direct electrodeposition on Ti–20Zr–5Ta–2Ag alloy with proven performance, and Naproxen integration to achieve multifunctionality—corrosion protection and local drug delivery. The kinetics of drug embedding and release were investigated, establishing an action mechanism and supporting this paper’s novelty.

The aim of this study was to develop and investigate Naproxen-doped polypyrrole films electrochemically deposited from a NADES electrolyte on Ti–20Zr–5Ta–2Ag alloy. Specifically, we prepared the alloy substrate and NADES–pyrrole–Naproxen electrolyte, electrodeposited the coatings under potentiostatic and galvanostatic conditions, characterized their surface and composition, assessed wettability and electrochemical stability in simulated body fluid, and evaluated Naproxen release kinetics by spectrophotometry and mathematical modelling.

The present study will be further complemented in the future with more bioinspired research, characterizing advanced materials with potentially greater performance in bioapplications.

## 2. Materials and Methods

All 73Ti-20Zr-5Ta-2Ag alloy samples were produced by levitation melting of high-purity Ti, Zr, Ta and Ag in a cold-crucible furnace under an inert argon atmosphere to avoid oxidation and to ensure the high temperatures required for Ta melting and compositional homogeneity. The resulting disks (20 mm diameter, 2 mm thickness) were then ground sequentially with SiC papers from P800 to P3600 (Buehler, Lake Bluff, IL, USA), ultrasonically cleaned for 10 min each in acetone, ethanol and distilled water, and air-dried. Uncoated disks served as controls.

Simulated body fluid (SBF, pH 7.4) was prepared following the Kokubo procedure [38] using analytical-grade salts from Sigma Aldrich (St. Louis, MO, USA) and ultrapure water (Millipore Direct Q 3UV, Merck, Molsheim, France, 18.2 MΩ·cm^−1^).

Natural deep eutectic solvent (NADES) was formulated by heating choline chloride (ChCl, 99%, Sigma Aldrich) with lactic acid in a 1:2 molar ratio to about 85 °C under continuous stirring until a homogeneous liquid formed; after cooling to room temperature, 0.5 M pyrrole (Py, 98%, Sigma Aldrich) and 10 mM Naproxen were added under stirring.

Using this NADES as an ionic liquid electrolyte, electropolymerization of polypyrrole (PPy) and PPy–Naproxen (Py–NAP, Naproxen EP Reference Standard, Sigma Aldrich) coatings was carried out in a single-compartment glass cell with an AutoLab 40 potentiostat/galvanostat (Radiometer Analytical SAS, Lyon, France), employing the alloy disks as working electrodes, a high-surface-area platinum counter electrode, and silver-wire quasi-reference electrodes. Cyclic voltammetry and chronoamperometry were used to monitor coating growth. For deposition, the applied potentials and currents were selected based on preliminary cyclic voltammetry experiments, ensuring efficient pyrrole oxidation and stable polymer growth while avoiding over-oxidation and degradation of the polymer. After deposition, samples were rinsed with ultrapure water and ethanol and air-dried.

Surface morphology was examined by optical microscopy using an AmScope ME520 Series Infinity-Corrected Darkfield Polarizing Metallurgical Microscope 50×–2500× Magnification with 18MP Camera (AmScope, Irvine, CA, USA) and by scanning electron microscopy (SEM) on a Nova NanoSEM 630 equipped with an EDX analyzer (FEI Company, Hillsboro, OR, USA). Chemical structures were probed by Fourier-transform infrared spectroscopy (FTIR; Perkin-Elmer, Shelton, WA, USA) over 4000–600 cm^−1^ at 4 cm^−1^ resolution (30 scans per sample).

Wettability was quantified by static contact-angle measurements of SBF droplets (3–5 µL via Hamilton syringe) on five distinct regions per specimen using a CAM 100 compact contact-angle meter (KSV Instruments, Espoo, Finland), reporting the mean of the measurements.

Protective performance of the PPy and PPy–NAP coatings in SBF was assessed electrochemically in a three-electrode cell (Pt sheet counter electrode, Ag/AgCl reference) by measuring open-circuit potential, electrochemical impedance spectroscopy at open-circuit (10 mV AC amplitude, 100 kHz–50 mHz), and potentiodynamic polarization (Tafel plots).

Finally, drug-release kinetics from TiZrTaAg–PPy–NAP samples were studied by immersion in 40 mL SBF, with 3 mL aliquots withdrawn at predetermined intervals (and replaced with fresh SBF) and Naproxen quantification at 230 nm by UV–Vis spectrophotometry (UV1720, UVISON Technologies, London, UK) to calculate cumulative release. It should be noted that the release profiles were calculated without applying corrections for possible polymer degradation or reduced drug activity in SBF. The amount of Naproxen loaded in the films was controlled by fixing its concentration in the NADES electrolyte (10 mM) and by terminating each deposition at a predetermined charge value (2 C). Since the total charge directly correlates with the amount of polymer deposited, this procedure ensured reproducible Naproxen incorporation.

For clarity and easy reference, the main symbols used throughout this manuscript are summarized in Table 1, including their names and units.

## 3. Results and Discussion

### 3.1. Pyrrole Embedded with Naproxen Electrodeposition

#### 3.1.1. Cyclic Voltammetry

**Figure 1:**
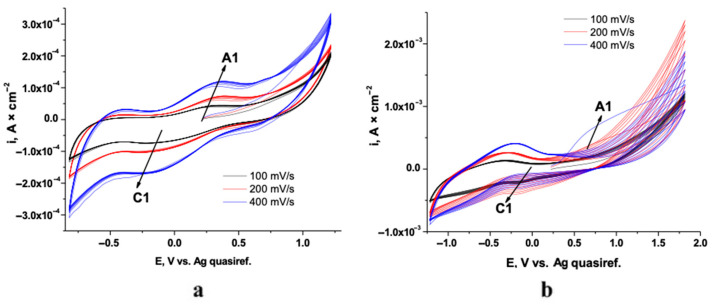
Cyclic voltammograms of the electrodeposition on Ti–Zr–Ta–Ag alloys recorded at various scan rates for (**a**) Ppy and (**b**) Ppy+NAP. Labelled peaks: A1—pyrrole oxidation/PPy nucleation–growth; C1—PPy reduction.

The initial phase involved examining the growth of PPy films electrosynthesized in ionic liquids on Ti–Zr–Ta–Ag alloys. Cyclic voltammetry was used to characterize the alloys’ electrochemical response in a natural deep eutectic solvent composed of choline chloride, lactic acid, and 0.5 M pyrrole. Figure 1 displays the resulting voltammograms recorded at various scan rates.

An anodic peak (A1) is observed, corresponding to monomer oxidation and the onset of polymer film formation. Notably, the anodic peak current density increases with successive scan cycles, indicative of a cumulative charge transfer and concomitant thickening of the polypyrrole layer. During the cathodic sweep, distinct reduction peaks emerge (C1), whose current densities likewise intensify as the number of cycles progresses. This cathodic process is attributable to the expulsion of chloride ions from the polymer matrix, reflecting the transition of polypyrrole from its oxidized to its neutral state. Thus, A1 corresponds to the oxidative polymerization of pyrrole leading to PPy film growth, while C1 reflects the reduction (de-doping) of the polymer with expulsion of counter-anions.

**Figure 2:**
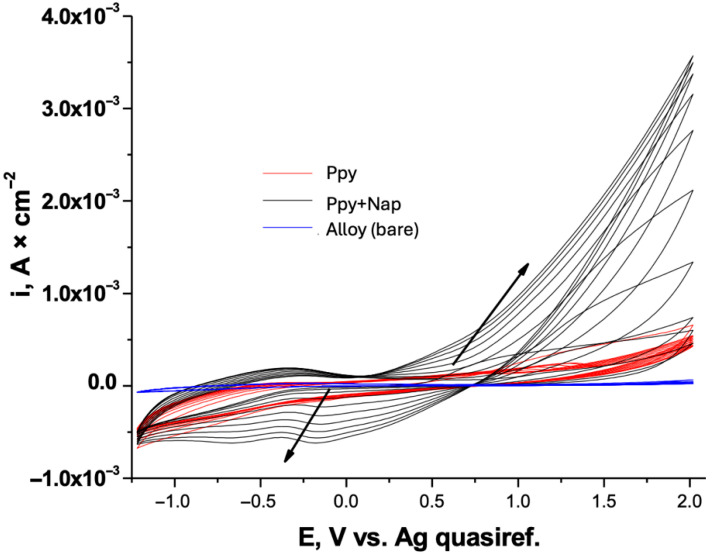
Comparative cyclic voltammograms recorded for Ti–Zr–Ta–Ag alloys in electrolyte without monomer, with pyrrole monomer, and with monomer plus drug at a scan rate of 200 mV s^−1^.

In order to evaluate the influence of the cycling potential window, a potential scan rate of 200 mV s^−1^ was employed. The corresponding results are presented in Figure 2.

In the absence of monomer, the alloy exhibits a featureless, near–zero current response, confirming its electrochemical inertness under these conditions. Upon addition of pyrrole, a pair of anodic and cathodic peaks emerges, indicative of monomer oxidation and subsequent polymer reduction. The inclusion of Naproxen further amplifies these redox features, particularly the anodic peak current, reflecting enhanced charge transfer associated with copolymer formation and drug incorporation.

#### 3.1.2. Controlled Deposition

**Figure 3:**
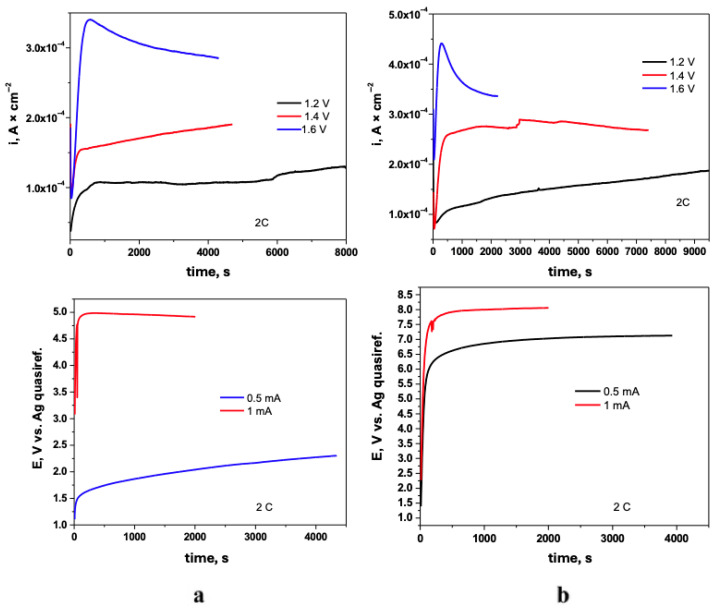
Chronoamperometric and chronopotentiometric profiles for PPy (**a**) and PPy+NAP (**b**) electrodeposition on TiZrTaAg alloys in NADES.

The electropolymerization of pyrrole on Ti–Zr–Ta–Ag was subsequently carried out in parallel by chronoamperometry and chronopotentiometry to produce films of pure PPy and PPy loaded with Naproxen. Chronoamperometric experiments were conducted at three fixed potentials, 1.2 V, 1.4 V, and 1.6 V versus an Ag quasi-reference electrode. For the chronopotentiometric depositions, constant currents of 0.5 mA and 1 mA were applied, and the corresponding potential–time profiles were recorded to monitor film growth under galvanostatic conditions. The resulting current–time transients are presented in Figure 3.

In the chronoamperometric plots, the initial surge in current, most pronounced at 1.6 V, reflects rapid nucleation and film formation, followed by a gradual decay as mass-transport limitations set in. Notably, the inclusion of Naproxen enhances the peak current density at each applied potential, suggesting that drug incorporation alters the polymerization kinetics and increases charge transfer.

In the chronopotentiometric traces (Figure 3b), the potential rapidly rises to stabilize at higher values for the 1 mA deposition, consistent with thicker films formed under greater current density. The Naproxen-containing system reaches plateau potentials more quickly and at slightly elevated levels compared to the drug-free polymer, indicating modified film conductivity and growth dynamics in the presence of the drug.

**Figure 4:**
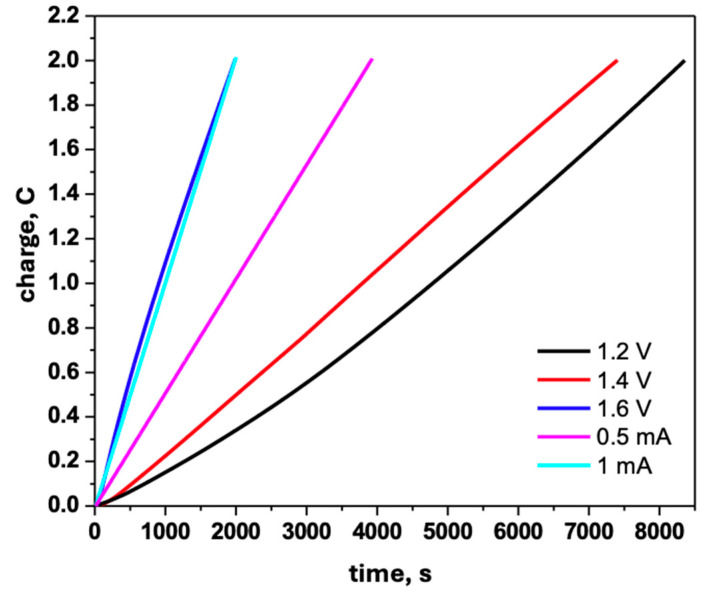
Comparative charge–time (Q-t) diagrams for PPy+Naproxen films electrodeposited under potentiostatic (1.2 V, 1.4 V, 1.6 V) and galvanostatic (0.5 mA, 1 mA) conditions.

Figure 4 presents the comparative charge–time (Q-t) profiles for PPy+Naproxen films electrodeposited on TiZrTaAg.

Curves recorded under potentiostatic conditions at 1.2 V, 1.4 V and 1.6 V exhibit linear charge accumulation, with steeper slopes at higher potentials reflecting faster polymer growth. For galvanostatic depositions, the Q-t traces are perfectly linear by construction, confirming constant current operation. The increased slope at 1 mA corresponds to a higher rate of charge transfer and, hence, thicker films over the same deposition time. These comparative diagrams demonstrate how the choice of deposition mode and applied parameter directly controls the amount of charge—and therefore film thickness—transferred during polymerization.

To ensure uniform film thickness, all samples were deposited to fixed charge values of 1.6 C, 2.0 C, and 2.2 C. Repeated experiments confirmed that both PPy and PPy–Naproxen coatings exhibit a high degree of reproducibility.

### 3.2. Surface Characterization

#### 3.2.1. Optical Microscopy

**Figure 5:**
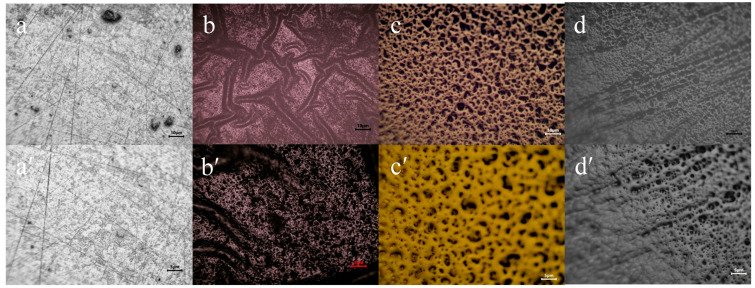
Optical micrographs of the TiZrTaAg alloy under different coating conditions: (**a**,**a′**) uncoated, (**b**,**b′**) coated with PPy, (**c**,**c′**) coated with PPy+Naproxen by potentiostatic deposition, and (**d**,**d′**) coated with PPy+Naproxen by galvanostatic deposition. Images a–d were taken at 10× magnification (scale bar = 10 μm), and images (**a′**–**d′**) at 20× magnification (scale bar = 5 μm).

Optical microscopy was used to investigate the surface morphology of TiZrTaAg alloy before and after coating with polypyrrole (PPy) and PPy doped with Naproxen (NAP), as shown in Figure 5.

Uncoated alloy (a,a′) exhibits a relatively smooth surface at both 10× and 20× magnifications, with faint scratching marks left from polishing. Deposition of a pure PPy film (b,b′) introduces a uniform, slightly granular texture, indicating complete coverage of the underlying substrate without large agglomerates. When Naproxen is incorporated potentiostatically (c,c′), the PPy+NAP layer displays a more pronounced, sponge-like network of interconnected nodules, suggesting that drug inclusion modifies nucleation and growth. Finally, the galvanostatically deposited PPy+NAP film (d,d′) appears denser and more compact than its potentiostatic counterpart, with fewer open voids and a smoother overall topology. These optical observations confirm that both deposition mode and the presence of Naproxen strongly influence film morphology and uniformity.

**Figure 6:**
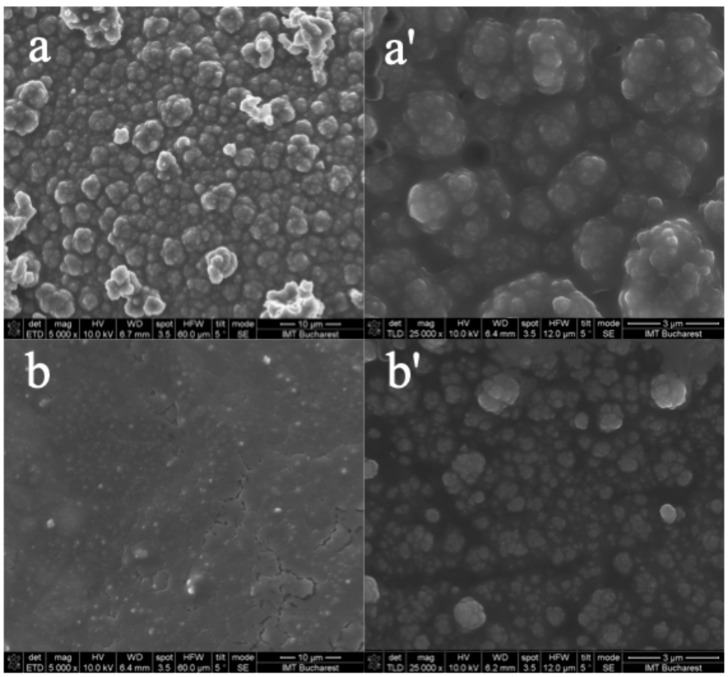
SEM images of PPy–NAP films deposited potentiostatically (**a**,**a′**) and galvanostatically (**b**,**b′**): **a**,**b**—magnification 5000×, scale bar 10 μm; **a′**,**b′**—magnification 25,000×, scale bar 3 μm.

In addition to the optical microscopy observations, the SEM images presented in Figure 6 offer a more detailed view of the coated surface morphology. The PPy–NAP film deposited potentiostatically (a,a′) reveals a network of well-defined nodules arranged in a sponge-like framework, confirming the interconnected granular texture seen under the optical microscope. By contrast, the film grown galvanostatically (b,b′) is markedly denser and more continuous, with sharply reduced porosity and a smooth topography.

While optical and SEM images clearly revealed morphological differences between potentiostatic and galvanostatic PPy–NAP coatings, quantitative measurements of film thickness and surface roughness were not included in the present study. These parameters are relevant for biomedical applications, as thickness influence long-term barrier performance and roughness can influence drug-release kinetics and cellular interactions.

#### 3.2.2. Fourier-Transformed Infrared (FTIR)

FTIR spectroscopy was employed to prove the chemical structure of the electrodeposited PPy–NAP coatings and to verify Naproxen incorporation. Given Naproxen’s carboxylic acid, aromatic, and methoxy functionalities, it exhibits strong, characteristic absorption bands in the mid-IR region (e.g., O–H stretch, C=O stretch, and aromatic C–H vibrations), which serve as clear markers of its presence [39].

**Figure 7:**
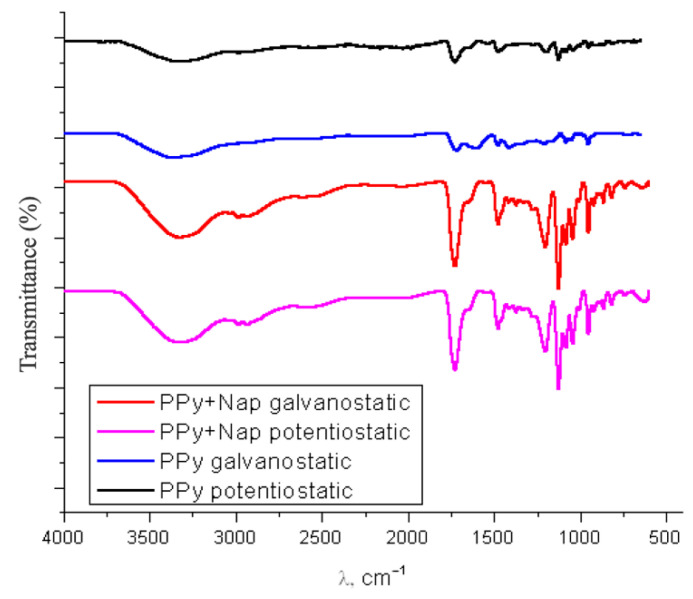
Overlaid FT-IR spectra (4000–600 cm^−1^) for Ti-20Zr-5Ta-2Ag+PPy and Ti-20Zr-5Ta-2Ag+PPy–NAP coatings.

To assess how Naproxen integrates into the PPy matrix, FTIR spectra were recorded for TiZrTaAg PPy-coated, and PPy–NAP-coated samples, as shown in Figure 7.

In the PPy-coated alloy, characteristic pyrrole peaks appear: a broad N–H stretch shoulder between 3400 and 3200 cm^−1^, the C=C stretch at ~1540 cm^−1^, the C–N stretch at ~1470 cm^−1^, and the pyrrole ring deformation around 957 cm^−1^. Once Naproxen is incorporated, new absorptions emerge at ~3250 cm^−1^ (adsorbed water), ~3020 cm^−1^ (aromatic C–H stretch), ~1550 cm^−1^ (carboxylic COO-stretch), ~1260 cm^−1^ (aromatic C–O–C stretch), ~1220 cm^−1^ (carboxyl C–O stretch), and ~745 cm^−1^ (out-of-plane aromatic C–H bend). These Naproxen bands are superimposed on the PPy signature peaks (e.g., C=C at ~1540 cm^−1^ and C–N at ~1470 cm^−1^), confirming drug presence.

#### 3.2.3. The Contact Angle

**Figure 8:**
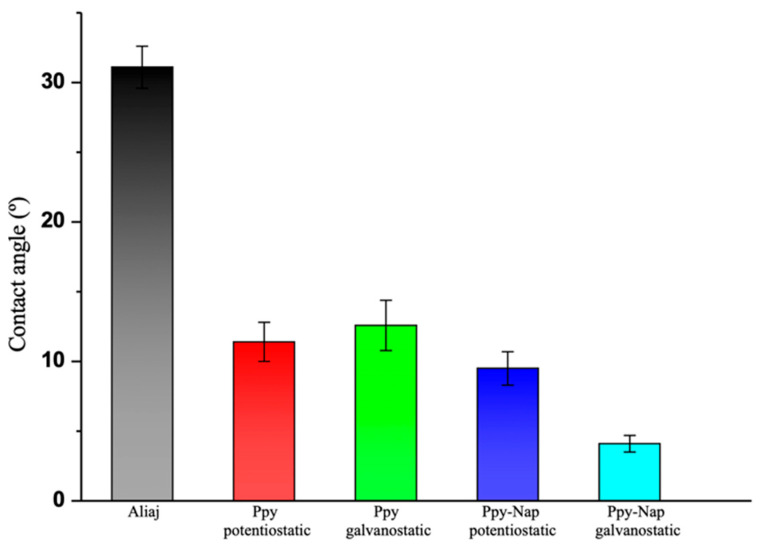
The contact angles for PPy or PPy+Naproxen films electrodeposited under potentiostatic (1.6 V) and galvanostatic (1 mA) conditions on TiZrTaAg samples, SBF droplets.

The hydrophilic or hydrophobic properties of a biomaterial, determined by its chemical composition and surface morphology, can be effectively characterized through contact-angle analysis. Since wetting behavior influences cell adhesion and proliferation on TiZrTaAg alloys, whether uncoated or coated with polypyrrole (PPy) or PPy doped with Naproxen (PPy–NAP), static contact angles were measured by placing droplets of simulated body fluid (SBF) onto five distinct locations on each sample. The mean contact angles and corresponding standard errors for bare TiZrTaAg, TiZrTaAg + PPy, and TiZrTaAg + PPy–NAP (both potentiostatic and galvanostatic) are presented in Figure 8.

The contact-angle measurements reveal a clear trend toward increasing hydrophilicity. The uncoated TiZrTaAg alloy exhibits a contact angle of 31.1 ± 1.5°, placing it at the lower end of a “moderately hydrophilic” regime, sufficient to allow some protein adsorption in a native-like conformation, but lacking optimized chemistry or roughness to fully support robust cell spreading. Coating with PPy alone reduces the contact angle to 11.4 ± 1.4° (potentiostatic, 1.6 V) and 12.6 ± 1.8° (galvanostatic, 1 mA), shifting the surface into a highly hydrophilic state. This rough, porous polymeric network presents abundant polar sites, encouraging serum proteins (e.g., fibrinogen and albumin) to adsorb without severe unfolding, conditions that generally enhance subsequent cell adhesion and proliferation [40].

Incorporating Naproxen into the PPy matrix drives the angle even lower, 9.5 ± 1.2° for potentiostatic PPy+NAP and 4.1 ± 0.6° for galvanostatic PPy+NAP—entering a superhydrophilic regime (<10°). Although a perfectly smooth, chemically inert superhydrophilic surface can sometimes maintain such a strong water layer that proteins remain trapped in their hydration shell and fail to tether to the solid interface, the PPy–NAP films are expected to avoid this pitfall in two ways. porous morphology, the polymer network is micro-/nano-rough, creating crevices where proteins can penetrate the hydration layer and anchor via van der Waals forces, hydrogen bonds, or electrostatic interactions, and complementary chemistry, Naproxen’s aromatic and polar functional groups (e.g., carboxylate and aromatic rings) provide specific binding sites that stabilize adsorbed proteins in biologically favorable conformations.

As a result, the superhydrophilicity of the PPy–NAP coatings might not impede protein binding. Instead, it might promote the rapid formation of a uniform, thin protein “conditioning film” while resisting non-specific bacterial adhesion. Taken together, all PPy-based coatings tested here exhibit significantly improved wetting compared to bare TiZrTaAg, and the Naproxen-doped variants, despite their superhydrophilicity, are expected to offer a chemically and morphologically favorable environment for protein adsorption, cell adhesion, and overall biocompatibility in biomedical applications.

### 3.3. Electrochemical Characterization

#### 3.3.1. Open-Circuit Potentials (OCPs)

OCPs measured over 20 min in SBF for samples coated with Ppy, and Ppy+NAP. The potential rapidly shifts toward more negative values during the first few seconds after immersion in SBF, then gradually drifts to a steady state within approximately 1200 s, regardless of whether the coating is pure PPy or PPy doped with Naproxen and independent of the deposition method. This behavior indicates that the polymer films formed on the TiZrTaAg substrate stabilize very quickly in physiological conditions. Specifically, all samples exhibit two distinct stages: an initial, rapid negative shift immediately after immersion, followed by a much slower movement toward a final negative value, culminating in a clearly defined plateau once equilibrium is reached.

**Figure 9:**
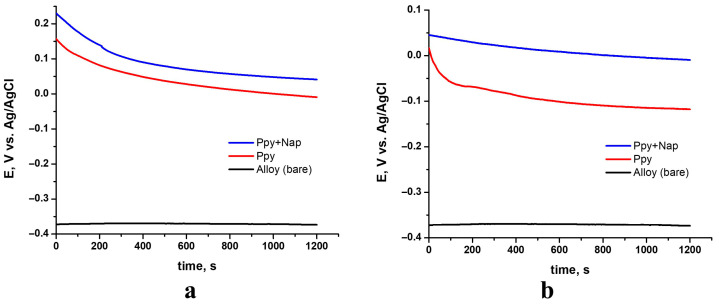
Comparative OCP vs. time in SBF for bare TiZrTaAg, TiZrTaAg+PPy, and TiZrTaAg+PPy–NAP at Q = 2 C. (**a**) potentiostatic 1.6 V; (**b**) galvanostatic 1 mA.

Figure 9 provides a comparative view of the stabilized OCP values for the uncoated alloy, PPy-coated, and PPy–NAP-coated samples under both potentiostatic (Figure 9a) and galvanostatic (Figure 9b) deposition conditions. By comparing these curves, the positive shift in equilibrium potential imparted by the polymer and drug-doped films relative to bare TiZrTaAg can be directly observed.

Both PPy and PPy–NAP coatings shift the open-circuit potential of TiZrTaAg to more positive values. The progressive positive shift from bare → PPy → PPy–NAP indicates increasingly effective barrier properties, with the Naproxen-doped films providing the highest corrosion-resistance potential in SBF.

#### 3.3.2. Tafel

Potentiodynamic polarization (Tafel) measurements were performed in SBF at a slow scan rate (2 mV s^−1^) to evaluate the corrosion behavior of bare TiZrTaAg and TiZrTaAg coated with PPy or PPy–NAP films.

**Figure 10:**
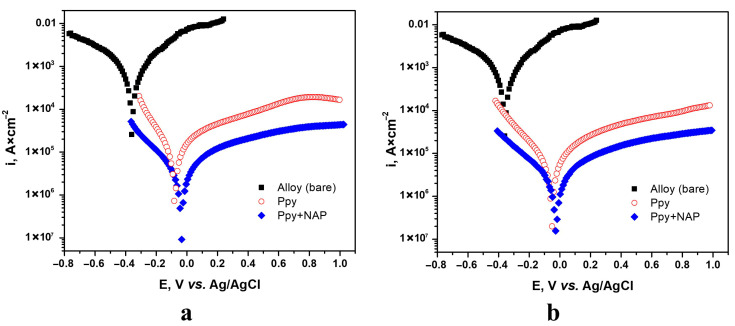
Comparative Tafel plots (Q = 2 C) in SBF for bare TiZrTaAg, TiZrTaAg+PPy, and TiZrTaAg+PPy–NAP: (**a**) potentiostatic (1.6 V, 2 C); (**b**) galvanostatic (1 mA, 2 C).

In order to directly compare the coated and uncoated alloy, Figure 10 overlays the Tafel curves for samples all grown to a fixed charge of 2 C, Figure 10a for potentiostatic films (1.6 V) and Figure 10b for galvanostatic films (1 mA).

Corrosion parameters were calculated relying on the current–potential characteristics within the Tafel region (i.e., the anodic and cathodic slopes) over a window of E = E_corr_ ± 250 mV as well as near the corrosion potential (E = E_corr_ ± 15 mV). During the determination of the Tafel slopes, a marked increase in the anodic slope was observed at a specific potential. This finding suggests that the electrode response is influenced by the presence of a certain amount of oxide on the metal surface, which likely induces a potential drop at the interface and alters either the metal–electrolyte interaction or the rate-determining step of the corrosion process.

The electrochemical parameters pertinent to the corrosion process have been determined using two methodologies: polarization resistance and Tafel slope extrapolation. By applying these rigorous and standardized methods, we obtain a precise and robust evaluation of the corrosion behavior for each system [41].

The primary parameters include corrosion potential (E_corr_), corrosion rate (V_corr_), corrosion current (i_corr_), polarization resistance (R_p_), coating porosity coefficient (P), and coating protection efficiency (P_i_), as detailed in Table 2. Based on these parameters, two critical parameters for assessing the corrosion susceptibility of coatings, the oxide protection efficiency (P_i_) and porosity coefficient (P) were calculated as well using Equations (1) and (2).

(1) $$P_{i}\left(\%\right)=\left[1-\left(\frac{i_{corr}}{i_{corr}^{0}}\right)\right]\times 100$$

where i_corr_—corrosion current density of the deposited layers; i^0^_corr_—corrosion current density of the substrate.

(2) $$P\left(\%\right)=\left(\frac{R_{ps}}{R_{p}}\right)\times 10^{-\left(\frac{\Delta E_{corr}}{\beta_{a}}\right)}$$

where P—total porosity of the deposited layer; R_ps_—polarization resistance of the uncoated substrate; R_p_—polarization resistance of the Ppy or Ppy-drug coated sample; Β_a_—anodic Tafel slope; ∆E_corr_—difference between the corrosion potentials of the uncoated and coated sample.

Depositing a PPy film on TiZrTaAg immediately reduces both the kinetic (current density) and thermodynamic (E_corr_) driving forces for corrosion. The polymer acts as a physical barrier that limits anodic dissolution and blocks aggressive ions in SBF. Incorporating Naproxen into the PPy matrix further enhances barrier properties—drug molecules fill polymer micropores, increase film compactness, and introduce additional hydrogen-bonding interactions that impede electrolyte penetration. As a result, PPy–NAP coatings consistently outperform pure PPy in all electrochemical metrics, and the close agreement between current densities derived from both Tafel-slope and polarization-resistance methods confirms the reliability of these findings.

Moreover, Tafel analysis shows that increasing the deposition charge at a constant potential of 1.6 V makes the PPy–NAP film significantly more protective. When the charge is raised from 1.6 C to 2.2 C, both anodic and cathodic Tafel slopes (βa,βc) increase while the corrosion current density (j_corr_) decreases. Consequently, the polarization resistance (R_p_) jumps from approximately 9,8 kΩ·cm^2^ to over 33,5 kΩ·cm^2^, and the corrosion rate falls from about 0.104 mm/year to below 0.025 mm/year, demonstrating that a thicker film provides a more effective barrier

Overall, the combined Tafel data demonstrate that PPy–NAP coatings can reduce TiZrTaAg’s corrosion rate from approximately 14 mm/year (bare) to below 0.03 mm/year (coated), with porosities on the order of 10^−5^%. Such performance is highly desirable for biomedical implants, where long-term corrosion resistance is critical.

#### 3.3.3. Electrochemical Impedance Spectroscopy (EIS)

**Figure 11:**
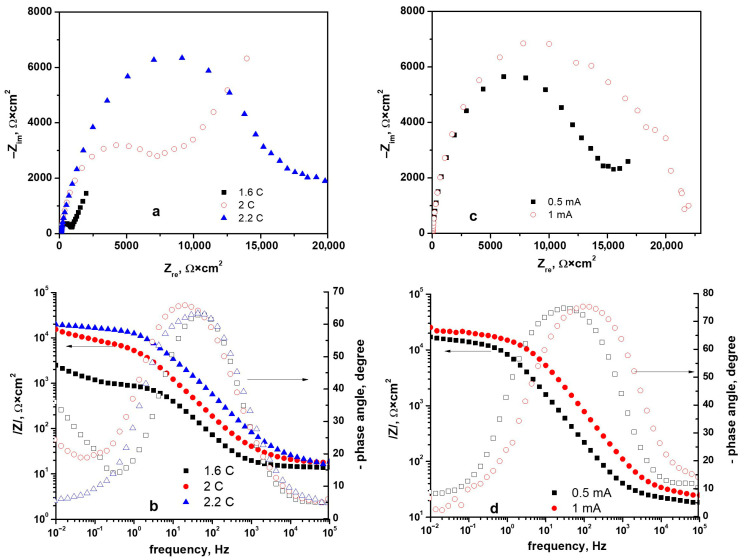
Nyquist (**a**,**c**) and Bode (**b**,**d**) plots in SBF for PPy–NAP coatings: potentiostatic (**a**,**b**) at 1.6 V (charges of 1.6 C, 2 C, and 2.2 C) and galvanostatic (**c**,**d**) at 0.5 mA and 1 mA (2 C).

EIS was employed to elucidate the barrier properties of the PPy–NAP coatings on TiZrTaAg in SBF. In Figure 11, the top row displays Nyquist plots, while the bottom row shows the corresponding Bode phase-frequency spectra. The left column corresponds to potentiostatic films at 1.6 V comparing three total charges, 1.6 C, 2 C and 2.2 C, for which R_ct_ rises from~0.77 kΩ·cm^2^ to~15 kΩ·cm^2^ (Table 3). The right column corresponds to galvanostatic films deposited to Q = 2 C at 0.5 mA vs. 1 mA, showing R_ct_ ≈ 12.2 kΩ·cm^2^ vs. 11.5 kΩ·cm^2^ and R_coat_ ≈ 10.4 × 10^3^ Ω·cm^2^ vs. 11.9 × 10^3^ Ω·cm^2^. These data confirm that increasing potentiostatic charge dramatically enhances both charge-transfer and coating resistance, while higher galvanostatic current yields a slightly denser film with elevated interfacial resistance.

The Nyquist plots for the PPy–NAP films in Figure 11a each display two capacitive semicircles—one at high frequency and one at low frequency—while the corresponding Bode phase spectra in Figure 11b show a prominent high-to-mid-frequency phase peak and a secondary maximum at lower frequencies. As the deposition charge at a constant potential of 1.6 V increases from 1.6 C to 2.0 C to 2.2 C, the diameter of the high-frequency semicircle in the Nyquist plots grows significantly, indicating progressively thicker, more resistive films. Concurrently, the low-frequency phase peak shifts to lower frequencies as the charge increases, reflecting slower interfacial processes in the denser coatings.

**Figure 12:**
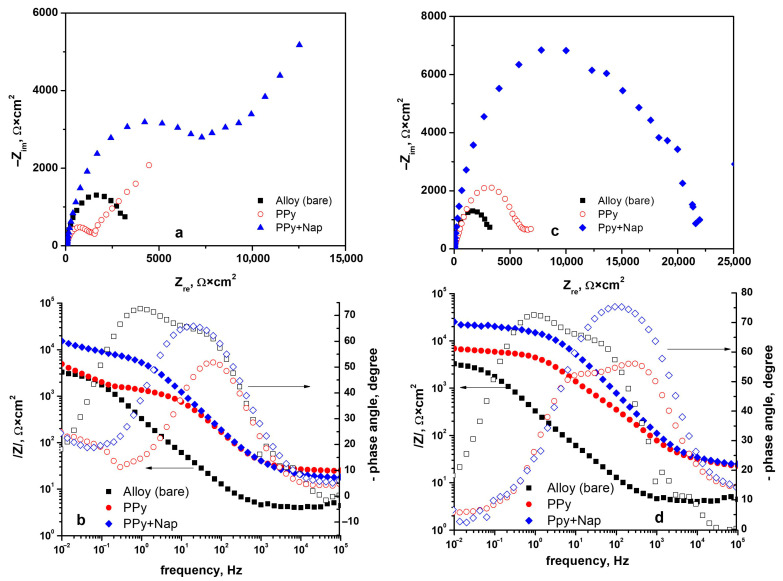
Comparative Nyquist (**a**,**c**) and Bode (**b**,**d**) plots in SBF for bare TiZrTaAg and TiZrTaAg coated with PPy or PPy+NAP, prepared in NADES: left panels (**a**,**b**)—chronoamperometric (potentiostatic at 1.6 V, 2 C) deposits; right panels (**c**,**d**)—chronopotentiometric (galvanostatic at 1 mA, 2 C) deposits.

To better compare the behavior of polymer coatings with and without incorporated drug, Figure 12 presents comparative impedance spectra that also include the uncoated TiZrTaAg substrate.

**Figure 13:**
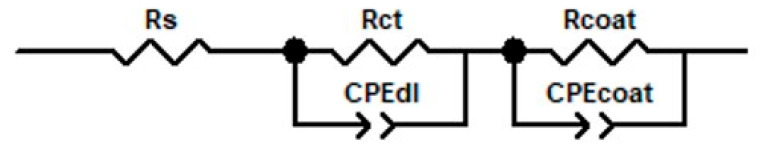
Equivalent electrical circuit model used to fit the EIS data.

The equivalent electrical circuit model used to fit the experimental results is shown in Figure 13, and the parameters extracted using this model are presented in Table 3.

**Table 3:** Equivalent circuit fitting parameters obtained from EEC modelling.

Material	Electropolymerizing Parameters	R_s_, Ω × cm^2^	R_ct_, KΩ × cm^2^	CPE_dl_—T, µF × cm^−2^	CPE_dl_—P	R_coat_, Ω × cm^2^	CPE_coat_—T, F × cm^−2^	CPE_coat_—P	Chi-Squared (χ^2^)
**Alloy**	-	4.29	3.39	640	0.84	19.25	0.86 × 10^−3^	0.87	5.1 × 10^−3^
**PPy/Alloy**	2 C; 1.6 V	25.1	1.35	36.6	0.787	4280	1.44 × 10^−3^	0.766	4.1 × 10^−3^
	2 C; 1 mA	19.6	6.02	32	0.718	7.43 × 10^9^	3.19 × 10^−3^	0.572	1.7 × 10^−3^
	1.6 C; 1.6 V	22.75	1.11	28.2	0.898	6.41 × 10^5^	1.75 × 10^−3^	0.638	1.6 × 10^−4^
**PPy+NAP** **/Alloy**	2 C; 1.6 V	23.37	6.42	26.2	0.871	2.59 × 10^4^	0.39 × 10^−3^	0.68	4.8 × 10^−4^
	1.6 C; 1.6 V	13.84	0.77	57.8	0.887	1.43 × 10^11^	2.5 × 10^−3^	0.655	1.1 × 10^−3^
	2.2 C; 1.6 V	24.4	14.98	8.92	0.836	9.3 × 10^9^	4.44 × 10^−4^	0.59	1 × 10^−3^
	2 C; 1 mA	24.33	11.53	4.16	0.944	11.86 × 10^3^	5.4 × 10^−5^	0.684	6.3 × 10^−3^
	2 C; 0.5 mA	18.68	12.2	16	0.918	10.36 × 10^3^	5.75 × 10^−4^	0.562	5.3 × 10^−3^

For all samples analyzed, the chi-squared (χ^2^) values remain between 1.6 × 10^−4^ and 6.3 × 10^−3^, indicating that the proposed equivalent circuit model reproduces the experimental EIS data with high fidelity.

Even the bare TiZrTaAg substrate exhibits a measurable charge-transfer resistance (R_ct_ ≈ 3.4 kΩ·cm^2^), but once a PPy film is deposited, R_ct_ changes dramatically depending on deposition mode and charge. For example, a PPy layer formed at 2 C/1 mA already shows R_ct_ ≈ 6.0 kΩ·cm^2^, roughly double that of the bare alloy, indicating a substantial barrier to electron transfer at the alloy/film interface. As the deposition charge, R_ct_ increases, reflecting progressively thicker, more compact PPy films.

When Naproxen is introduced into the PPy matrix, this barrier effect becomes even more pronounced. A PPy–NAP film deposited at 1.6 C/1.6 V already yields R_coat_ on the order of 1.4 × 10^11^ Ω·cm^2^, compared with only 6.4 × 10^5^ Ω·cm^2^ for a similar PPy coating. In parallel, R_ct_ increases from about 0.77 kΩ·cm^2^ (1.6 C at 1.6 V) up to nearly 15 kΩ·cm^2^ (2.2 C at 1.6 V). Higher deposition charge and the choice of galvanostatic mode (e.g., 2 C at 0.5 mA or 1 mA) lead to R_ct_ values in the 11–12 kΩ·cm^2^ range. This trend shows that Naproxen incorporation and thicker films progressively hinder charge transfer at the alloy/film interface—especially in the 2.2 C and galvanostatic samples, where R_ct_ reaches its maximum.

Constant phase-element (CPE) parameters corroborate this picture: the double-layer CPE capacitance (CPEdl–T) decreases markedly from ~640 µF·cm^−2^ (bare alloy) to single-digit µF·cm^−2^ values for the thickest, drug-doped coatings, while CPEdl–P approaches unity, indicating an increasingly ideal capacitive behavior at the alloy/film interface.

Together, these impedance and CPE trends demonstrate that (1) PPy alone forms a competent barrier, (2) adding Naproxen drastically enhances both coating integrity and interface resistance, and (3) higher deposition charge or potential yields near-perfect ionic blockage. In practical terms, these PPy–NAP coatings are effectively impermeable to aggressive species in SBF, making them exceptionally well suited for long-term corrosion protection of biomedical implants.

### 3.4. Drug Release

**Figure 14:**
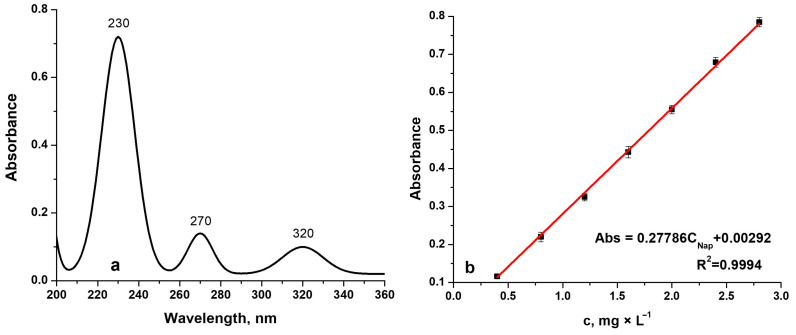
(**a**) UV–Vis spectrum of 20 mg/L sodium Naproxen in SBF (absorbance peaks at 230 nm, 270 nm, 320 nm); (**b**) calibration curve at 230 nm (0–50 mg/L Naproxen; n = 3).

Drug-release experiments were conducted by immersing PPy–NAP–coated TiZrTaAg samples in SBF at 37 °C. Figure 14a shows the UV–Vis spectrum of 20 mg/L sodium Naproxen in SBF, where absorbance maxima appear at 230 nm, 270 nm, and 320 nm. A calibration curve (Figure 14b) was then constructed by measuring absorbance at 230 nm for standard Naproxen concentrations (0–50 mg/L, n = 3), yielding a linear correlation (R^2^ > 0.999).

Two sets of Naproxen-loaded coatings were prepared under identical fixed charge, 2 C, but different deposition modes: a potentiostatic film (1.6 V) formed by chronopotentiometry and a galvanostatic film (1 mA) formed by chronoamperometry. Both coatings were synthesized from a NADES electrolyte (ChCl–lactic acid) containing 0.5 M pyrrole and 10 mM sodium Naproxen. The cumulative release fraction *R*(*t*) (in %) was calculated as:

(3) $$R\%=100*\frac{q(t)}{q_{0}}$$

where *q*_0_—initial Naproxen load in the film; *q*(*t*)—the amount released at time *t*.

**Figure 15:**
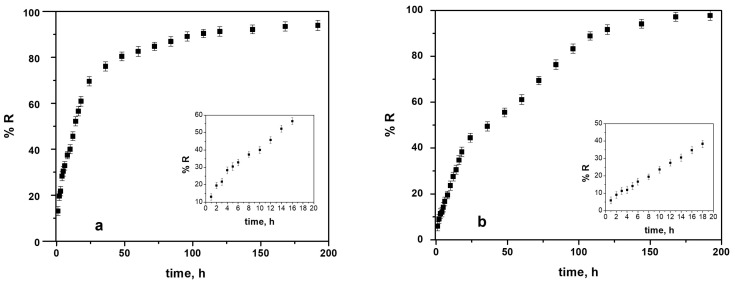
Cumulative Naproxen release profiles in SBF at 37 °C from PPy–NAP coatings (fixed charge = 2 C): (**a**) potentiostatic (1.6 V); (**b**) galvanostatic (1 mA).

Figure 15 compares the two release profiles. In the potentiostatic sample (Figure 15a), an initial burst releases approximately 21% of the drug in the first 3 h. Release remains rapid over the next 15 h, reaching 60.5% at 18 h and about 70% at 24 h. Thereafter, the film enters a sustained-release regime: over half of the incorporated Naproxen is released by 18 h, followed by a slower, quasi-linear release over the next 100 h. After 192 h, the cumulative release fraction reaches 93.93%.

By contrast, the galvanostatic coating (Figure 15b) exhibits a smaller initial burst (12% at 3 h) and slower release up to 18 h (38%). Between 24 h and 144 h, however, release accelerates from 44% to 94%, reflecting a delayed but more pronounced diffusion phase. Ultimately, the galvanostatic film achieves 97.78% cumulative release after 192 h. These results demonstrate that PPy–NAP coatings can deliver drug over extended periods, with potentiostatic deposition favoring a higher early-stage burst and galvanostatic deposition producing a more uniform, protracted release.

Mathematical modelling of Naproxen release from PPy coatings can provide deeper insight into the mechanisms governing drug diffusion. Most empirical and semi-empirical release models used in pharmaceutical research are derived from Fick’s law of diffusion [42,43,44]. In this study, the in vitro release data were fitted to five kinetic models, zero-order, first-order, Higuchi, Peppas–Korsmeyer, and Hixson–Crowell, to determine which best describes the release behavior. The resulting kinetic parameters, including the rate constant k, correlation coefficient R^2^, and diffusion exponent n, are summarized in Table 4.

Analysis of the kinetic parameters for each mathematical model shows that the highest correlation coefficient (R^2^) was obtained with the Peppas–Korsmeyer model, indicating that this model most accurately describes Naproxen release. The Korsmeyer–Peppas equation is a simple semi-empirical expression frequently used to characterize drug-release kinetics in pharmaceutical research [45]. It is given by:

(4) $$R(t)=k*t^{n}$$

where *R*(*t*)—the cumulative fraction of drug released at time *t*; *k*—kinetic constant (which also incorporates structural and geometric features of the polymer-drug system); *n*—the diffusion exponent that characterizes the release mechanism.

The Peppas–Korsmeyer model serves as a decision-making tool for identifying dominant transport mechanisms. For n = 0.5, release is governed by Fickian diffusion. For 0.5 < n < 1.0, anomalous (non-Fickian) transport occurs, indicating a combination of diffusion and polymer-chain relaxation (or erosion). For n= 1.0, zero-order release applies, meaning the drug is released at a constant rate independent of time.

In practice, Equation (4) should be applied only to the first ~60% of total release (R < 60 %). In our case, the potentiostatic PPy–NAP coating yields n = 0505, indicating predominantly Fickian diffusion with minimal polymer relaxation, whereas the galvanostatic coating gives n = 0.667, signifying transport that includes both diffusion and polymer-matrix erosion.

The kinetic constants k are 13.29 h^−1^ for the potentiostatic film and 7.73 h^−1^ for the galvanostatic film, suggesting a relatively faster drug release from coatings formed at constant potential compared to those formed at constant current.

Practically, the parameters k and n indicate coating performance under physiological conditions. The kinetic constant k reflects both diffusion rate and polymer structure, with higher values corresponding to faster, burst-like release. The diffusion exponent n defines the transport mechanism: values near 0.5 suggest diffusion through water-filled pores, while values between 0.5 and 1.0 indicate combined diffusion and polymer relaxation. Thus, a coating with n ≈ 0.5 release mainly by diffusion, whereas a coating with n ≈ 0.67 involve both diffusion and relaxation.

## 4. Conclusions

This study has demonstrated the electrochemical deposition of polypyrrole and Naproxen-doped polypyrrole films from a natural deep eutectic solvent onto a Ti20Zr5Ta2Ag alloy.

Optical microscopy revealed that pure polypyrrole deposition produced a uniform granular film fully covering the substrate. Potentiostatic incorporation of Naproxen generated a porous, sponge-like architecture of interconnected nodules, clearly visible in high-resolution SEM as an open framework. In contrast, galvanostatic deposition yielded a markedly denser, more compact coating with a smooth topology and minimal porosity in the SEM images, underscoring the enhanced film uniformity.

FTIR analysis further confirmed Naproxen’s integration into the polymer matrix, as evidenced by characteristic peaks for its carboxylate and aromatic functional groups.

Contact-angle measurements showed a marked increase in surface hydrophilicity: the uncoated alloy (≈31°) became more wettable with PPy coatings (≈11–13°) and reached superhydrophilic values (≈4–10°) when Naproxen was included. The resulting porous morphology and complementary chemistry are expected to support protein adsorption and cellular adhesion without excessive protein denaturation.

Electrochemical tests in simulated body fluid indicated increased corrosion protection from both PPy and PPy–NAP films. Compared to the bare substrate’s corrosion current density of 754 µA/cm^2^, PPy reduced this to 4.5–7.0 µA/cm^2^ and PPy–NAP to 1.3–5.5 µA/cm^2^. Polarization resistance increased from 0.063 kΩ·cm^2^ (bare) to 5.9–11.2 kΩ·cm^2^ (PPy) and 9.8–37.8 kΩ·cm^2^ (PPy–NAP). Nyquist plots and low-frequency impedance values from EIS corroborated these improvements, indicating enhanced barrier properties with drug incorporation.

Naproxen release in SBF at 37 °C followed a two-stage profile: an initial burst of 12–21% within 3 h, 38–70% by 18–24 h, and 94–98% by 144–192 h. Analysis via the Peppas–Korsmeyer model yielded diffusion exponents of n ≈ 0.505 for potentiostatic films (Fickian transport) and n ≈ 0.667 for galvanostatic films (anomalous transport), with rate constants k reflecting faster release under potentiostatic conditions.

In summary, PPy–NAP films on Ti20Zr5Ta2Ag combine improved electrochemical stability with controlled drug-release behavior and enhanced surface wettability. These attributes suggest their suitability for further investigation as multifunctional coatings in biomedical implant applications.

The present work will be continued with a focus on biomimetic approaches to strengthen its relevance for biomedical applications. Future efforts should focus on in vitro assays on relevant cell lines to evaluate cytotoxicity, protein adsorption, and cellular adhesion. These investigations will be essential to confirm the multifunctional potential of the coatings for implant applications.

## Figures and Tables

**Table 1:** Notation of the main parameters used in this study.

Symbol	Name	Unit
E_corr_	Corrosion potential	mV
i_corr_	Corrosion current density	µA·cm^−2^
K_g_	Corrosion rate (gravimetric)	g·m^−2^·h^−1^
V_corr_	Corrosion rate (electrochemical)	mm·year^−1^
R_p_	Polarization resistance	kΩ·cm^2^
P	Coating porosity coefficient	%
P_i_	Coating protection efficiency	%
R_ct_	Charge-transfer resistance (from EIS)	kΩ·cm^2^
R_coat_	Coating resistance (from EIS)	Ω·cm^2^
R_s_	Solution resistance (from EIS)	Ω·cm^2^
CPE_dl_-T	Constant phase element (double layer) magnitude	µF·cm^−2^
CPE_dl_-P	CPE exponent (double layer)	–
CPE_coat_-T	Constant phase element (coating) magnitude	F·cm^−2^
CPE_coat_-P	CPE exponent (coating)	–
q_0_	Initial Naproxen load in the film	mg (relative)
q(t)	Amount of Naproxen released at time *t*	mg
R(t)	Cumulative release fraction	%
n	Diffusion exponent (Korsmeyer–Peppas model)	–
k	Kinetic constant (drug release models)	h^−1^

**Table 2:** Polarization parameters corresponding to the PPy or PPy+NAP-coated TiZrTaAg alloy.

Material			Tafel Slope Method			Polarization Resistance Method		Pi (%)	P (%)
	Electropolymerization Parameters	E_corr_, mV	i_corr_, µA × cm^−2^	Kg, g × m^2^ × h^−1^	V_corr_, mm × year^−1^	R_p_, kΩ × cm^2^	i_corr_, µA × cm^−2^		
**Alloy**		−373 ± 0.5	754 ± 1.2	10.65 ± 0.3	14.22 ± 0.1	0.063 ± 0.02	722 ± 1.5	-	-
**PPy/Alloy**	2 C; 1.6 V	−69 ± 0.3	6.81 ± 0.02	0.096 ± 0.005	0.128 ± 0.03	6.80 ± 0.7	6.04 ± 0.05	99.09 ± 0.01	3.85 × 10^−4^ ± 0.03
	2 C; 1 mA	−59 ± 0.3	4.5 ± 0.04	0.064 ± 0.002	0.085 ± 0.001	11.15 ± 0.5	4.21 ± 0.01	99.40 ± 0.01	2.11 × 10^−4^ ± 0.02
	1.6 C; 1.6 V	−19 ± 0.2	7.02 ± 0.08	0.099 ± 0.003	0.132 ± 0.005	5.9 ± 0.3	6.97 ± 0.02	99.07 ± 0.01	2.62 × 10^−4^ ± 0.01
**PPy–NAP/Alloy**	2 C; 1.6 V	−34 ± 0.3	1.65 ± 0.03	0.023 ± 0.001	0.031 ± 0.001	29.7 ± 0.5	1.35 ± 0.01	99.57 ± 0.02	6.1 × 10^−5^ ± 0.005
	1.6 C; 1.6 V	−39 ± 0.2	5.52 ± 0.04	0.078 ± 0.003	0.104 ± 0.01	9.8 ± 0.1	5.14 ± 0.02	99.27 ± 0.005	1.95 × 10^−4^ ± 0.02
	2.2 C; 1.6 V	125 ± 0.9	1.35 ± 0.01	0.019 ± 0.001	0.025 ± 0.002	33.5 ± 0.5	1.35 ± 0.02	99.82 ± 0.01	1.02 × 10^−5^ ± 0.04
	2 C; 1 mA	−46 ± 0.3	1.26 ± 0.02	0.018 ± 0.001	0.024 ± 0.001	37.8 ± 0.5	1.25 ± 0.02	99.83 ± 0.005	5.44 × 10^−5^ ± 0.01
	2 C; 0.5 mA	37 ± 0.1	2.96 ± 0.04	0.042 ± 0.002	0.056 ± 0.002	17.5 ± 0.2	2.72 ± 0.02	99.61 ± 0.02	4.93 × 10^−5^ ± 0.02

**Table 4:** Kinetic parameters from empirical model fitting.

Deposition Mode	Zero Order		First Order		Higuchi		Hixson–Crowell		Peppas–Korsmeyer		
	$\%R=k_{0}t$		$ln(100-\%R)=k_{1}t$		$\%R=k_{2}\surd t$		$\sqrt[{3}]{100-\%R}=k_{3}t$		$\%R=k_{4}\sqrt[{n}]{t}$		
	***R* ^2^**	***k*_0_**	***R* ^2^**	***k*_1_**	***R* ^2^**	***k*_2_**	***R* ^2^**	***k*_3_**	***R* ^2^**	***k*_4_**	***n***
**Potentiostatic (1.6 V)**	0.9797	2.62	0.9851	0.0428	0.9885	12.82	0.963	0.053	0.9929	13.29	0.505
**Galvanostatic (1 mA)**	0.9931	1.88	0.9956	0.024	0.9734	8.51	0.9931	1.65	0.9973	7.73	0.667

## Data Availability

The raw data supporting the conclusions of this article will be made available by the authors upon request.
